# Demographic and Psychological Factors Associated with Feelings of Antibiotic Entitlement in New Zealand

**DOI:** 10.3390/antibiotics7030082

**Published:** 2018-09-05

**Authors:** Carol H. J. Lee, Pauline Norris, Isabelle M. Duck, Chris G. Sibley

**Affiliations:** 1 School of Psychology, University of Auckland, Auckland 1010, New Zealand; c.sibley@auckland.ac.nz; 2 School of Pharmacy, University of Otago, Dunedin 9016, New Zealand; pauline.norris@otago.ac.nz; 3 Westgate Medical Centre, Auckland 0614, New Zealand; isabelle.duck@wgmc.co.nz

**Keywords:** antibiotic entitlement, antibiotic prescription, New Zealand, personality traits

## Abstract

Patients’ expectations of being prescribed antibiotics can have an important influence on inappropriate prescribing. Therefore, it is important to understand the drivers of patients’ antibiotic expectations. The 2015/16 New Zealand Attitudes and Values Study measured sense of entitlement to antibiotics in a nationally representative sample of New Zealanders (*n* = 13,484). Participants were asked to rate their agreement with the statement “If I go to my doctor/GP with a minor illness (e.g., sore throat, cough, runny nose, etc.), I think that I should be prescribed antibiotics by default.” Eighty percent of participants showed low feelings of antibiotic entitlement, while 18.5% exhibited moderate and 3.7% high feelings of entitlement. People of ethnic minority, lower socio-economic status, and with diabetes expressed higher expectations of being prescribed antibiotics. This may be partially based on a higher risk of rheumatic fever or other complications. Men, religious people, those with lower educational attainment and self-rated health, but greater psychological distress and feelings of control over their health exhibited higher feelings of antibiotic entitlement. Those high on Extraversion, Conscientiousness, and Narcissism, but low on Agreeableness and Openness, also showed greater feelings of entitlement. Our findings help identify key characteristics of those more likely to express inappropriate expectations of antibiotic prescription.

## 1. Introduction

Antibiotic resistance is a serious and growing threat to global public health [1,2]. Due to the excessive and inappropriate use of antibiotics, it is becoming more difficult and expensive to treat common infections, and there is rising concern that certain infections may become impossible to treat in the future [1]. Among the general public, many people are unaware of, or have superficial knowledge about, antibiotic resistance and mistakenly believe that antibiotics can help treat self-limiting viral infections [3,4]. Thus, some expect to be prescribed antibiotics when consulting their doctor about cold or flu symptoms [4]. Such inappropriate patient expectations have important implications, as doctors are more likely to feel pressured to prescribe antibiotics when they perceive that patients expect or demand antibiotics [5,6]. Prior findings thus highlight the importance of ensuring that patients have accurate knowledge and appropriate expectations of antibiotics. 

### 1.1. Antibiotics in the Context of New Zealand

Over recent years, New Zealand has witnessed a rise in antibiotic use and the number of antibiotic-resistant infections [2,7]. The rate of antibiotic consumption increased by around 49% from 2006 to 2014, with dispensing rates being highest among young children and Pacific peoples [7]. Data from the Growing up in New Zealand study suggests that most children (97%) have been exposed to antibiotics and received an average of 1.9 antibiotic courses per year by the age of five years [8]. Antibiotic dispensing was found to be higher among Māori and Pacific children, and children living in the most deprived areas [8]. Additionally, a study conducted in Gisborne found that antibiotic use was greater among women, young individuals, and the elderly [9]. 

Rheumatic fever remains a problem in New Zealand, almost entirely amongst Māori and Pacific populations [10]. Because of this, campaigns to reduce antibiotic use are tempered by the need to increase use when rheumatic fever is a possible outcome of a sore throat. In New Zealand, the sore-throat guideline recommends that doctors endorse a lower threshold for throat swabbing and prescribing antibiotics for patients with a higher risk of streptococcal sore throat [10]. This includes those with a personal, family, or household history of rheumatic fever, and those who satisfy two or more of the criteria of being of ‘Māori or Pacific ethnicity’, ‘aged 3–35 years’, and ‘living in crowded conditions or lower socioeconomic areas.’ The Ministry of Health has also increased efforts to increase public awareness about and reduce the incidence of rheumatic fever [10]. National and community campaigns have been implemented to raise awareness about the link between sore throats and rheumatic fever and encourage doctor visits for children with sore throats in high risk groups. Before the campaign, a study had found that Māori individuals, especially those in rural areas, were less likely to receive antibiotic prescriptions [9]. However, the campaign to reduce rheumatic fever is likely to have led to a substantially increased use amongst people from Māori and Pacific communities.

Currently, little is known about group disparities in feelings of entitlement to being prescribed antibiotics for minor illnesses such as a cold or viral infection. Given the actual difference in prescription guidelines, it is reasonable to expect that Māori or Pacific individuals with lower socioeconomic status (SES) have greater expectations about receiving antibiotics. This expectation is likely based on their higher need for and likelihood of being prescribed antibiotics rather than feelings of entitlement. Moreover, it is also important to consider one’s health condition as this may influence their susceptibility to infections and complications, and likelihood of being prescribed antibiotics [11,12,13]. That is, patients who are systematically unwell, have pre-existing co-morbidities (e.g., heart, lung disease), or otherwise have a higher risk of developing complications are more likely to require and therefore receive antibiotics for a minor infection [13].

In contrast to those with a higher risk of rheumatic fever or health complications, greater expectations about receiving antibiotics among low-risk healthy individuals may arise from an inappropriate sense of entitlement to antibiotics. This sense of entitlement may stem from a complex range of factors, including the lack of or inaccurate antibiotic knowledge, previous experiences of illnesses or antibiotic consumption, and one’s health-related beliefs. With the growing problem of antibiotic resistance, it is especially important to identify the characteristics and underlying psychological drives of those who exhibit this inappropriate sense of entitlement.

### 1.2. Self-Rated Health

Subjective health beliefs have an important influence on the types of medication people choose to use. A Brazilian study found that those with poor self-rated health were more likely to keep medication at home, take medication frequently, and have received a medical prescription in the previous two weeks [14]. Indeed, this may be because those with poor self-rated health are more likely to have health conditions that require medication. However, this trait has further been associated with an increased use of potentially inappropriate medication [15], and likelihood of self-medicating with antibiotics [3]. These findings suggest that those with poor subjective health tend to show a strong tendency to take and rely on medication, even when it may not be necessary or suitable for their condition. Hence, if those with poor subjective health believe that antibiotics can treat a cold or perhaps simply desire medication, they may exhibit greater expectations about receiving antibiotics for a minor illness.

### 1.3. Feeling of Control over Health Outcomes

One’s feeling of control over their health is also a key determinant of health-related behaviors. Those who believe they can control their health outcomes through their own behavior are more likely to take actions to change their negative health behaviors, such as smoking or drinking [16,17]. In a similar way, those with greater feelings of control may be more likely to seek ways to regain their health when they have a minor illness. If these individuals have had previous experiences of recovering from a cold after taking antibiotics or inaccurate beliefs about antibiotics, they may regard seeking antibiotics as a sensible health behavior aimed at relieving their cold symptoms. Such mistaken beliefs may further lead them to express increased expectations about receiving antibiotics when consulting their doctor about a cold or flu.

### 1.4. Personality Traits

Personality traits are closely associated with one’s health status and attitudes (see Appendix A for definitions of Big-five personality traits). For instance, Extraversion, Agreeableness, and Conscientiousness has been linked with higher, while Neuroticism has been linked with lower, levels of subjective wellbeing [18]. Conscientious is a particularly well-established predictor of good health and greater engagement in numerous positive health behaviors [19]. In regard to adherence to antibiotic therapy, Axelsson found that Agreeableness and Conscientiousness were positive predictors, and Neuroticism was a negative predictor, of medication compliance [20].

To our knowledge, previous studies have not examined the direct association between the Big-five personality traits and feelings of entitlement to receiving antibiotics when visiting their doctor with a cold or viral infection. Furthermore, little is known about the association between one’s level of Narcissism and expectations about receiving antibiotics. Narcissism is a trait characterized by beliefs that one is superior and entitled to more things in life than others [21]. Narcissistic individuals tend to show confidence in their judgement, even when they do not have accurate knowledge [21], and are less trusting of others [22]. Based on these findings, it is likely that Narcissistic individuals exhibit greater confidence in their inaccurate knowledge about antibiotics and feel more entitled to receiving antibiotics when they believe they need them. 

Extending on past research, the current study uses a nationally representative sample of New Zealand adults to examine the distribution of one’s belief that they should be prescribed antibiotics by default when consulting their doctor with a minor illness (e.g., sore throat, cough, runny nose). We investigate how a range of demographic and psychological characteristics, such as ethnicity, household income, subjective health beliefs, the Big-five personality traits, and Narcissism, may influence one’s feeling of entitlement to antibiotics. Further, we assess and control for the effect of various health factors that may influence one’s susceptibility to catching a cold or experiencing complications following a minor illness. This includes psychological distress, sleep duration, Body Mass Index (BMI), smoking status, and diagnosis with chronic illnesses such as heart diseases or diabetes. Findings from this study will help identify those more likely to express an inappropriate sense of antibiotic entitlement and provide a framework for future research on interventions for promoting the sensible use of antibiotics.

## 2. Results

This study uses the seventh wave (2015/16) of the NZAVS (Total *n* = 13,944), in which 13,484 participants answered the item on antibiotic entitlement. Participants rated their degree of agreement to the statement: “If I go to my doctor/GP with a minor illness (e.g., sore throat, cough, runny nose, etc.), I think that I should be prescribed antibiotics by default” on a scale of 1 (strongly disagree) to 7 (strongly agree; unweighted *M* = 1.76, *SD* = 1.26, after applying sample weighting *M* = 1.89, *SD* = 1.38).

### 2.1. Participants

Participants had a mean age of 50.79 (*SD* = 13.9) and median household income of $90,000. Around 63% of our sample were female and 37% were male. Seventy eight percent were employed, 75.6% were parents, and 74.8% were in a serious romantic relationship. Roughly 90% of participants identified as being New Zealand European, 12% as being Māori, 3.1% as being of the Pacific, 3.9% as being Asian, and 1.7% as being of another ethnicity (categories are not mutually exclusive). The average BMI of participants was 27.36, with 8.3% being smokers and 29.6% having a limiting disability or illness for six or more months. Lastly, 4.5% reported being diagnosed with diabetes and 4.8% with heart disease.

### 2.2. Antibiotic Entitlement Groups

As shown in Table 1, the following scale ranges were used to describe *low entitlement* (1–2), *moderate entitlement* (3–5), and *high entitlement* (6–7). Most participants exhibited low entitlement, but around one fifth showed moderate to high levels of entitlement. (See Appendix A for table depicting demographic and psychological characteristics of entitlement groups).

### 2.3. Demographic Correlates

A range of demographic and psychological variables were simultaneously included in a multiple regression predicting people’s feelings of antibiotic entitlement on a continuous scale of 1 (low) to 7 (high). The coefficients are interpreted based on whether a predictor variable is associated with an increase or decrease in one’s feelings of antibiotic entitlement.

As seen in Table 2, our results indicated that men (*b* = 0.171, *SE* = 0.023, *p* < 0.001), those who had lower educational attainment (*b* = −0.037, *SE* = 0.005, *p* < 0.001), and those who were religious (*b* = 0.149, *SE* = 0.021, *p* < 0.001) exhibited higher feelings of entitlement to antibiotics. Those with a lower (log) household income (*b* = −0.095, *SE* = 0.018, *p* < 0.001), lower SES (*b* = −0.004, *SE* = 0.001, *p* < 0.001), and higher deprivation (*b* = 0.008, *SE* = 0.004, *p* = 0.044) also expressed greater feelings of entitlement. Compared to Europeans (reference category), Māori (*b* = 0.173, *SE* = 0.038, *p* < 0.001), Asian (*b* = 0.451, *SE* = 0.068, *p* < 0.001), and Pacific (*b* = 0.542, *SE* = 0.084, *p* < 0.001) individuals showed greater expectations about receiving antibiotics. Age; parental, partner, and employment status; and region of residence were not significantly associated with one’s sense of antibiotic entitlement.

### 2.4. Psychological and Health Related Correlates

Those with higher levels of self-rated health (*b* = −0.088, *SE* = 0.013, *p* < 0.001) exhibited lower, while those who felt more in control of their health (*b* = 0.054, *SE* = 0.012, *p* < 0.001) and had higher psychological distress (*b* = 0.014, *SE* = 0.004, *p* = 0.002) showed increased feelings of entitlement. Smokers (*b* = 0.126, *SE* = 0.047, *p* = 0.007), those with a higher BMI (*b* = 0.010, *SE* = 0.002, *p* < 0.001) and those with a diabetes diagnosis (*b* = 0.141, *SE* = 0.065, *p* = 0.029) also showed greater expectations about receiving antibiotics. On the other hand, those who indicated having a limiting disability or illness that lasted six or more months (*b* = −0.091, *SE* = 0.025, *p* < 0.001) and with asthma (*b* = −0.067, *SE* = 0.032, *p* = 0.037) exhibited lower expectations.

Those high on Extraversion (*b* = 0.066, *SE* = 0.010, *p* < 0.001) and Conscientiousness (*b* = 0.043, *SE* = 0.011, *p* < 0.001) showed greater, while those high on Agreeableness (*b* = −0.075, *SE* = 0.013, *p* < 0.001) and Openness to Experience (*b* = −0.106, *SE* = 0.010, *p* < 0.001) showed lower, feelings of entitlement. Additionally, those high on Narcissism (*b* = 0.146, *SE* = 0.010, *p* < 0.001) expressed greater feelings of entitlement (Honesty-Humility was omitted from analysis due to multicollinearity with Narcissism). While most demographic and psychological predictors showed trivial effects, Narcissism had an unstandardized beta greater than 0.01 (*β* = 0.148), indicating a strong association with feelings of antibiotic entitlement.

## 3. Discussion

The present study used a nationally representative sample of New Zealand adults to investigate the distribution and identify demographic and psychological correlates of peoples’ feelings of entitlement to antibiotics. Most New Zealanders showed low feelings of entitlement (77.9%), while 18.5% exhibited moderate and 3.7% high feelings of entitlement. Although only a small subset of the population expressed high entitlement, a fair proportion appear to show some degree of expectation about receiving antibiotics when visiting their doctor with a minor illness. This can be linked to the lack of knowledge about the correct use of antibiotics among the general public [23,24], and highlights the importance of increasing public awareness about the dangers of antibiotic resistance.

Although most effects were rather trivial, our results revealed significant demographic differences in feelings of antibiotic entitlement. Men, religious people, and those with lower educational attainment exhibited greater feelings of entitlement. People of Māori, Asian, or Pacific ethnicity, with a lower household income and SES, and higher deprivation, also expressed greater expectations about receiving antibiotics. As the sore throat guidelines recommend a lower threshold for prescribing antibiotics to people of Māori or Pacific ethnicity and lower SES [10], and our question specifically mentions the term ‘sore throats’, it is not surprising to find that these groups show higher expectations about receiving antibiotics. Given their increased risk of rheumatic fever [10], greater expectations among these groups cannot be regarded as mere feelings of entitlement, but could rather be viewed as appropriate expectations based on accurate knowledge about their need for antibiotics.

As one’s health condition influences their risk of developing complications and likelihood of being prescribed antibiotics [11,12,13], we controlled for and assessed the effect of various health factors on people’s feelings of antibiotic entitlement. Having a high BMI or diabetes diagnosis, and being a smoker were associated with increased feelings of entitlement. As these factors have been linked with negative health outcomes and/or a greater risk of serious health complications [11,12,25], doctors may be more likely to prescribe antibiotics to individuals who exhibit these characteristics. However, it is important to further investigate why these particular factors may be showing significant effects, independent of other key demographic and health variables. Interestingly, participants who indicated having a long-term illness/disability or asthma showed decreased feelings of entitlement. The effect of asthma is unexpected as common cold viruses have been linked with exacerbations of asthma, which is often and inappropriately treated with antibiotics [26,27]. On the other hand, as the item on long-term illness/disability only asked about whether participants had an illness or disability that limited them for at least six months, it is difficult to identify what specific illness or disability may be driving this effect.

In terms of psychological factors, we found that lower self-rated health and higher psychological distress were associated with greater feelings of antibiotic entitlement. As high psychological distress can negatively affect the immune system, it can increase one’s susceptibly to infectious diseases, as well as the severity and duration of an illness [28,29]. Thus, those with higher levels of stress may be more likely to desire medication to help them recover from their pro-longed and severe cold symptoms. Our findings also suggest that poor self-rated health is not only related to a greater use of medication [14,15], but may also induce greater feelings of entitlement to antibiotics. Those with poor self-rated health may perceive their minor illness to be more severe and believe that they cannot easily recover from a cold unless they take antibiotics.

Generally, feeling more in control of one’s health is regarded as a positive trait linked with an increased likelihood of taking actions to change negative health behaviors [16,17]. However, paradoxically, this trait was linked with increased feelings of antibiotic entitlement. Beliefs that antibiotics can treat the common cold or previous experiences of using antibiotics to treat such illnesses may lead to perceptions that taking antibiotics is a positive way of acting to recover from a cold. Little et al. [30] found that receiving immediate antibiotic prescriptions for sore throats tends to increase one’s belief about the effectiveness of antibiotics in treating sore throats and the likelihood of consulting a doctor in the future. Such positive beliefs about antibiotics may extend to an increased feeling of entitlement to antibiotics; a resource deemed necessary to improve their health outcomes. Alternatively, simply being able to access medication when needed may increase one’s feelings of control over their health outcomes. This raises the possibility that greater feelings of accessibility to antibiotics rather than beliefs about their ability to self-care for infections using antibiotics may be driving this effect.

Of the Big-five personality traits, all factors but Neuroticism showed a significant association to people’s feelings of antibiotic entitlement. Those high on Extraversion and Conscientiousness but low on Agreeableness and Openness to Experience exhibited greater feelings of entitlement. The effect of Agreeableness is not surprising, as this trait has been negatively related to psychological entitlement [31]. Interestingly, Conscientiousness individuals, who tend to adopt positive health behaviors and have a high health status [19], were found to show higher feelings of antibiotic entitlement. Due to the widespread misconception that antibiotics can treat viral infections [23,24], Conscientious individuals may mistakenly believe that taking antibiotics for a cold is a positive health behavior and therefore aspire to adopt this behavior. Such mistaken beliefs may be driving the paradoxical effect of both Conscientiousness and feelings of control over one’s health. Hence, raising awareness about the appropriate use of antibiotics may potentially reverse these paradoxical effects.

Lastly, Narcissistic individuals were found to exhibit a greater sense of antibiotic entitlement. While this may be an expected finding as Narcissism was measured using items from a psychological entitlement scale, it is a novel finding that one’s belief that they deserve more things in life extends to a greater sense of entitlement to antibiotics in a medical context. As Narcissistic individuals tend to exhibit overconfidence [21], they are more likely to feel confident about their knowledge about antibiotics, and thus exhibit a greater demand for antibiotics when they believe it is an effective treatment for their illness. In contrast to the trivial effects of other demographic or psychological predictors, Narcissism showed a particularly strong association with feelings of antibiotic entitlement. This suggests that Narcissism may be a key trait driving patient expectations about antibiotics.

Findings from this study provide a snapshot of general attitudes towards antibiotics among New Zealanders and help identify key characteristics of those more likely to exhibit inappropriate expectations about receiving antibiotic prescriptions. They also provide an important framework for future research aimed at informing interventions for altering patient attitudes towards antibiotics. By increasing research on the role and utility of personality and psychological factors, we may be able to identify more effective doctor-patient communication strategies to ensure doctors can adequately respond to patients who demand antibiotics. That is, if doctors can identify the personality traits or health beliefs of a patient, they may be able to utilize this knowledge to deliver antibiotic information in a way most suitable and effective for changing the views of that patient. For instance, by emphasizing the dangers of incorrect or excessive use of antibiotics, doctors may be able to persuade Conscientious individuals that taking antibiotics unnecessarily is a negative health behavior and consequently reduce their desire for antibiotics.

### Limitations

Limitations of this study include the use of cross-sectional data and a single-item to measure people’s feelings of entitlement to antibiotics. This means we are unable to imply causality from our results or identify the specific reasons why those from different groups may be exhibiting high or low feelings of entitlement. We could not examine whether inaccurate antibiotic knowledge was driving higher feelings of entitlement, or if feelings of entitlement can be linked with greater antibiotic consumption. As some studies have identified discrepancies between one’s health-related beliefs and actual health behavior [32,33], it is also unknown whether participants would actually act in line with their responses when consulting a doctor in reality. Additionally, there may have been disparities in the way people perceive the severity of “sore throats”, “runny noses”, and “coughs”, which were listed as examples of a minor illness in our survey item. Some participants may also have interpreted the question to include possible streptococcal infection, which does indeed require antibiotic treatment. Future studies should aim to develop more sophisticated measures of feelings of antibiotic entitlement, and further assess New Zealanders’ knowledge about antibiotics and their actual level of antibiotic use.

## 4. Materials and Methods

### 4.1. Sampling Procedure

The New Zealand Attitudes and Values Study (NZAVS) is a longitudinal panel study with a probability sample of New Zealand adults. This study is reviewed by The University of Auckland Human Participants Ethnics Committee every three years and has most recently been approved for 5-September-2017 until 3-June-2021 (Reference Number: 014889). The initial Time 1 (2009) NZAVS recruited participants by randomly selecting samples from the New Zealand electoral roll (response rate: 16.6%) [34]. A booster sample was later recruited at Time 3 (2011) through an unrelated survey posted on the website of a major New Zealand newspaper. Further booster samples were recruited from the 2012 and 2014 Electoral Roll in subsequent Time periods. This study uses data from the seventh wave (2015/16) of the NZAVS (*n* = 13,484). The validity of the NZAVS in monitoring changes in New Zealanders’ political attitudes over time has been well-demonstrated [35]. 

### 4.2. Measures

Participants’ feeling of antibiotic entitlement was measured using the item: “If I go to my doctor/GP with a minor illness (e.g., sore throat, cough, runny nose, etc.), I think that I should be prescribed antibiotics by default,” which was rated on a scale of 1 (strongly disagree) to 7 (strongly agree). This item was developed for the NZAVS in consultation with medical professionals.

Participants were asked to provide their gender, relationship, smoking and employment status, date of birth, and annual household income. Ethnicity was measured using the standard New Zealand Census item, in which participants could indicate each ethnic group they identified with. Education was coded into an eleven-level ordinal variable (0 = no qualification to 10 = doctorate degree). Deprivation was measured using the 2013 New Zealand Deprivation Index, which uses census information to assign a decile-rank index from 1 (least deprived) to 10 (most deprived) to each meshblock unit [36]. SES was measured using the New Zealand socio-economic index [37]. 

Personality traits were measured using the Mini-IPIP6 [38], which assesses the six major dimensions of personality using four-item subscales rated from 1 (very inaccurate) to 7 (very accurate). Narcissism was measured using items from the psychological entitlement self-report measure (e.g., “I deserve more things in life”) [39].

Self-rated health was measured using three marker items (using seven-point scales) from the Short-Form Health Questionnaire (e.g., “I expect my health to get worse”) [40]. Feeling of control over health was measured using Likert items from the multidimensional health locus of control scale (e.g., “I am in control of my health”) [41]. The Kessler-6 scale was used to measure people’s level of psychological distress [42]. Final Kessler-6 scores were obtained by adding participants’ responses to six items, which were measured on a scale of 0 (low distress) to 4 (high distress). Participants rated their level of satisfaction with their “access to healthcare when they need it (e.g., doctor, GP)” on a scale of 0 (completely dissatisfied) to 10 (completely satisfied). They also indicated whether they had been diagnosed with a range of illnesses including heart disease, diabetes, asthma, and high blood pressure, as well as whether they had a “health condition or disability that limited them, and that has lasted for 6+ months.”

### 4.3. Statistical Analyses

A multiple regression examining the association between various demographic and psychological variables with New Zealanders’ level of antibiotic entitlement was conducted on Mplus. The continuous item used to measure people’s feeling of entitlement (on a scale of 1–7) was used as the outcome variable, and all predictor variables were simultaneously included in the regression. Missing data for exogenous variables were estimated using Rubin’s procedure for multiple imputation. Final parameter estimates were obtained by averaging 10,000 imputed datasets (thinned using every 200th iteration) generated based on information in the existing data and random elements. Descriptive statistics were calculated using SPSS after applying sample weighting.

### 4.4. Sample Weighting Procedure

To estimate representative population proportions, the NZAVS uses a post-stratification weight that corrects for sample bias in gender and ethnic group identification [43]. As the Time 4 (2012) sample included regional booster samples, weights from Time 4 onwards include regional information. The weighting procedure for this study was based on population demographic data from the 2013 New Zealand Census, with sample weights being determined by one’s region of residence, gender, and ethnicity. Weights for men and women from each of the four primary ethnic groups were calculated separately.

## 5. Conclusions

The current study investigated New Zealanders’ level of agreement with the statement that one should be prescribed antibiotics by default when they visit their doctor with a minor illness. Most New Zealanders exhibited low feelings of antibiotic entitlement, but around one fifth expressed moderate-to-high feelings of entitlement. People belonging to an ethnic minority, those who smoked, had lower SES and high psychological distress, those with a higher BMI, and those with diabetes expressed greater expectations about receiving antibiotics. Having asthma and a long-term illness/disability were unexpectedly linked with decreased expectations. Men, religious people, and those with lower educational attainment and self-rated health exhibited higher feelings of entitlement. Those high on Extraversion and Narcissism but low on Agreeableness and Openness to Experience showed a greater sense of entitlement. Paradoxically, Conscientious individuals and those who felt more in control of their health also exhibited greater feelings of entitlement. Taken together, our findings increase insight into the psychological factors that influence one’s expectations about receiving antibiotics and provide guidance for future research on interventions to reduce the over-prescription of antibiotics.

## Figures and Tables

**Table 1:** Frequencies and percentages of participants within each antibiotic entitlement group.

Group	Frequencies		Percentages	
	Weighted (*n* = 13,464)	Unweighted (*n* = 13,484)	Weighted	Unweighted
*High Entitlement*	492	373	3.7%	2.8%
(ratings of 6–7)				
*Moderate Entitlement*	2489	2156	18.5%	16.0%
(ratings of 3–5)				
*Low Entitlement*	10483	10955	77.9%	81.2%
(ratings of 1–2)				

Note: Standard NZAVS sample weighting applied. See methods for details on weighting procedure.

**Table 2:** Regression predicting level of entitlement to receiving antibiotic prescriptions by default when visiting their doctor with a minor illness.

	*b*	*SE*	Lower 95% CI	Upper 95% CI	*β*	*t*	*p*-Value
Log (income)	−0.095	0.018	−0.131	−0.060	−0.074	−5.325	0.000 **
NZ Deprivation (0–10)	0.008	0.004	0.000	0.016	0.018	2.013	0.044 *
Socio-economic status	−0.004	0.001	−0.005	−0.002	−0.042	−4.267	0.000 **
Education (0 low to 10 high)	−0.037	0.005	−0.047	−0.028	−0.082	−7.801	0.000 **
Gender (0 women, 1 men)	0.171	0.023	0.125	0.217	0.065	7.276	0.000 **
Age	−0.002	0.001	−0.004	0.000	−0.018	−1.652	0.099
Māori (0 no, 1 yes)	0.173	0.038	0.099	0.247	0.044	4.587	0.000 **
Pacific (0 no, 1 yes)	0.542	0.084	0.377	0.706	0.074	6.461	0.000 **
Asian (0 no, 1 yes)	0.451	0.068	0.318	0.584	0.069	6.629	0.000 **
Religious (0 no, 1 yes)	0.149	0.021	0.107	0.191	0.058	6.965	0.000 **
Parent (0 no, 1 yes)	0.040	0.027	−0.013	0.094	0.014	1.481	0.139
Partnered (0 no, 1 yes)	−0.016	0.028	−0.071	0.039	−0.005	−0.564	0.573
Employed (0 no, 1 yes)	0.007	0.029	−0.050	0.064	0.002	0.236	0.813
Urban area (0 rural, 1 urban)	0.042	0.023	−0.003	0.086	0.016	1.841	0.066
Self-rated health	−0.088	0.013	−0.114	−0.062	−0.080	−6.643	0.000 **
Health locus of control	0.054	0.012	0.031	0.077	0.046	4.584	0.000 **
Healthcare access	−0.012	0.006	−0.023	0.000	−0.019	−1.939	0.052
BMI	0.010	0.002	0.006	0.014	0.048	4.753	0.000 **
Smoking status (0 no, 1 yes)	0.126	0.047	0.034	0.219	0.028	2.687	0.007 **
Disability or illness 6+ months	−0.091	0.025	−0.139	−0.042	−0.033	−3.644	0.000 **
High Cholesterol (0 no, 1 yes)	0.016	0.030	−0.042	0.074	0.005	0.553	0.580
High blood pressure (0 no, 1 yes)	0.012	0.030	−0.048	0.071	0.004	0.388	0.698
Hearth disease (0 no, 1 yes)	0.047	0.058	−0.066	0.161	0.008	0.812	0.417
Diabetes (0 no, 1 yes)	0.141	0.065	0.014	0.268	0.023	2.179	0.029 *
Asthma (0 no, 1 yes)	−0.067	0.032	−0.131	−0.004	−0.017	−2.089	0.037 *
Sleep duration (average night)	−0.004	0.010	−0.024	0.016	−0.004	−0.371	0.711
Kessler-6 score (stress level)	0.014	0.004	0.005	0.022	0.042	3.162	0.002 **
**Narcissism**	**0.146**	**0.010**	**0.126**	**0.166**	**0.148**	**14.516**	0.000 **
Extraversion	0.066	0.010	0.047	0.085	0.061	6.887	0.000 **
Agreeableness	−0.075	0.013	−0.100	−0.050	−0.057	−5.920	0.000 **
Conscientiousness	0.043	0.011	0.021	0.064	0.034	3.817	0.000 **
Neuroticism	0.004	0.012	−0.020	0.027	0.003	0.292	0.770
Openness	−0.106	0.010	−0.126	−0.086	−0.093	−10.500	0.000 **

Notes: * *p* < 0.05, ** *p* < 0.01. Predictor with standardized beta coefficient greater than 0.10 is bolded. Model fit statistics: R^2^ = 0.155, AIC = 42,381.019, BIC = 42,643.843. (Average *n* = 13,484).

## Appendix A

**Table A1:** Interpretation of Big-Five personality traits, including example traits, and likely adaptive benefit and costs resulting from high levels of each personality dimension (adapted from Sibley et al.) [38].

Factor	Interpretation	Example Traits	Likely Adaptive Benefits of High Levels (in Evolutionary History)	Likely Costs of High Level (in Evolutionary History)
Extraversion	Engagement in social endeavours	Sociability, leadership, exhibition	Social gains (friends, mates, allies)	Energy and time; risks from social environment
Agreeableness	Ingroup co-operation and tolerance; reciprocal altruism in HEXACO model	Tolerance, forgiveness, (low) quarrelsomeness	Gains from cooperation, primarily with ingroup (mutual help and nonaggression)	Losses due to increased risk of exploitation in short-term exchanges
Conscientiousness	Engagement in task-related endeavours	Diligence, organization, attention to detail	Material gains (improved use of resources), reduced risk	Energy and time; risks from social environment
Neuroticism (low Emotional Stability)	Monitoring of inclusionary status and attachment relations; kin altruism in HEXACO model.	Anxiety, insecurity, (low) calmness	Maintenance of attachment relations; survival of kin in HEXACO model	Loss of potential gains associated with risks to attachment relations.
Openness to Experience	Engagement in ideas-related endeavours	Curiosity, imaginativeness, (low) need for cognitive closure and (low) need for certainty	Material and social gains (resulting from discovery)	Energy and time; risks from social and natural environment

**Table A2:** Demographic and psychological characteristics of low, moderate, and high entitlement groups.

	Age	Household Income	Narcissism	Gender		Ethnicity (% within Ethnic Group)			
				**Female**	**Male**	**European**	**Māori**	**Pacific**	**Asian**
Low entitlement	50.28 (13.89)	112,157.76 (94,064.42)	2.62 (1.29)	55.8%	44.2%	83.2%	68.9%	55.6%	65.8%
Moderate entitlement	48.73 (14.47)	91,911.96 (70,940.92)	3.30 (1.25)	48.2%	51.8%	14.8%	24.2%	33.0%	27.7%
High entitlement	49.81 (14.46)	79,287.57 (61,714.74)	3.86 (1.70)	50.9%	49.1%	2.0%	6.9%	11.3%	6.5%
	**Self-Rated Health**	**Belief about Health Controllability**	**Kessler-6 Score (Distress Level)**	**Disability or Illness for 6+ Months**	**Big-Five Personality Traits**				
					E	A	C	N	O
Low entitlement	5.15 (1.13)	5.08 (1.10)	4.79 (3.75)	28.5%	3.88 (1.17)	5.32 (0.96)	5.10 (1.01)	3.35 (1.12)	5.00 (1.10)
Moderate entitlement	4.87 (1.09)	5.05 (0.98)	5.84 (4.23)	26.6%	3.87 (1.05)	5.01 (0.90)	5.02 (0.95)	3.56 (1.03)	4.63 (1.00)
High entitlement	4.78 (1.40)	5.43 (1.16)	6.64 (5.35)	30.5%	4.09 (1.20)	5.00 (1.08)	5.11 (1.07)	3.61 (1.05)	4.62 (1.14)

Notes: Table reports percentages for categorical variables, and mean values for continuous variables (standard deviations in brackets). Narcissism, belief about health controllability, self-rated health, and Big-five personality traits were measured on scale of 1 (low) to 7 (high). Kessler risk scores range from 0 (lowest) to 24 (highest). Satisfaction with healthcare access was measured on scale of 0 (low) to 10 (high). ‘E’ = Extraversion, ‘A’ = Agreeableness, ‘C’ = Conscientiousness, ‘N’ = Neuroticism, ‘O’ = Openness. Standard NZAVS weighting variable applied.
